# Number of Nanoparticles per Cell through a Spectrophotometric Method - A key parameter to Assess Nanoparticle-based Cellular Assays

**DOI:** 10.1038/srep10091

**Published:** 2015-05-15

**Authors:** Juan D. Unciti-Broceta, Victoria Cano-Cortés, Patricia Altea-Manzano, Salvatore Pernagallo, Juan J. Díaz-Mochón, Rosario M. Sánchez-Martín

**Affiliations:** 1 Pfizer - Universidad de Granada - Junta de Andalucía Centre for Genomics and Oncological Research (GENYO), Parque Tecnológico de Ciencias de la Salud (PTS), Avenida de la Ilustración 114, 18016 Granada, Spain; 2 NanoGetic S. L. Parque Tecnológico Ciencias de la Salud (PTS), Avenida de la Innovación 1, Edificio BIC, 18016 Armilla – Granada (Spain); 3 DestiNAGenomica S.L. Parque Tecnológico Ciencias de la Salud (PTS), Avenida de la Innovación 1, Edificio BIC, 18016 Armilla – Granada (Spain); 4 Departamento de Química Farmacéutica y Orgánica. University of Granada, Campus de Cartuja s/n, 18017 Granada, Spain

## Abstract

Engineered nanoparticles (eNPs) for biological and biomedical applications are produced from functionalised nanoparticles (NPs) after undergoing multiple handling steps, giving rise to an inevitable loss of NPs. Herein we present a practical method to quantify nanoparticles (NPs) number per volume in an aqueous suspension using standard spectrophotometers and minute amounts of the suspensions (up to 1 μL). This method allows, for the first time, to analyse cellular uptake by reporting NPs number added per cell, as opposed to current methods which are related to solid content (w/V) of NPs. In analogy to the parameter used in viral infective assays (multiplicity of infection), we propose to name this novel parameter as *multiplicity of nanofection*.

Over the last decades, nanotechnologies have become essential tools in the biomedical field with a large number of products based on nanoparticles being found in the market^1^. Nanoparticles (NPs) from a wide range of different organic and inorganic materials are used in different biological and medical applications, such as diagnostic, non-invasive imaging and drug delivery to mention just a few^2^,^3^,^4^,^5^. Therefore, Engineered Nanoparticles (eNPs) are increasing their relevance in various fields, including targeted drug and gene delivery systems, biosensors and cancer treatment^6^,^7^,^8^,^9^,^10^,^11^,^12^,^13^,^14^,^15^. In particular, polystyrene eNPs have an extensive use as systems for *in vitro* applications such as cell-sorting, labelling and cellular delivery^16^,^17^.

Our research team, for instance, has developed protocols for the preparation of multifunctionalised cross-linked polystyrene NPs, with a size range of ~0.2–2 μm. These polystyrene NPs enable solid phase multistep chemistries and are compatible with different bio-orthogonal strategies^18^,^19^. Their chemical robustness allows conjugating onto them a large variety of biological and chemical cargoes to give rise to eNPs^20^ following multi-step solid-phase organic synthesis. These eNPs have been proven to be taken up by the majority of cell types studied so far while exhibiting no detrimental effect on cell viability^21^. Their cellular uptake depends on incubation time, particle size distribution and concentrations used in cell culture media^22^,^23^,^24^. To correlate eNPs with robust biological responses and to ensure the reproducibility and significance of the results, an accurate and reliable method for quantifying the number of eNPs used per assay is crucial. However, in most cell-based assays based on nanoparticles, number of cells plated and volume (microliters) of nanoparticle suspensions, with an estimated solid content (weight/volume percent), are the typical parameters reported. Since these assays fit well with particle collision models^25^, the optimal way of describing the protocol used in the assay would be by providing number of eNPs versus number of cells. Such a parameter would enable to assess eNP performances in different cell lines.

The usual manner of determining the nanoparticles number per microliter of suspension is based on the application of an equation where the values of the solid content and size are absolutely necessary (Supplementary Note S1). However, eNP preparations require numerous centrifugal steps with the consequent loss of nanoparticles, meaning an inevitable reduction of solid content from step to step. This issue would be addressed by enabling quantification of the number of eNPs per microliter of suspensions after each step, rather than referring necessary to the initially calculated solid content. This is even more relevant in multi-step eNP syntheses or high-throughput studies, because they are generally done at small scales, making solid content measurements very impractical. Consequently, this loss of NPs leads to an unknown eNPs concentration which is a major drawback in the development and understating of many assays and an important issue to obtain reproducible results from lab to lab.

Herein, we present a practical method to estimate the number of polystyrene NPs per microliter via optical light scattering using conventional spectrophotometers and tiny volumes. The new method is inspired in the common nephelometric methods used to estimate bacteria numbers in suspension. This method allowed us to use number of eNPs added per cell as a parameter for cell-based assays.

## Results and discussion

So far, NPs numbers have been only considered in phagocytosis assays to report how many particles per cell are phagocyted^26^,^27^. However, the need to obtain reproducible cellular uptake results using eNPs which underwent multiple steps to conjugate chemical and biological cargoes led us to generate an approximation method to calculate the number eNPs per microliter. Based on nephelometric principals, a practical method was developed by measuring the turbidity optical density at 600 nm (OD600), wavelength used for nephelometric-based bacterial counting, of polystyrene NP suspensions. Light going through NP suspensions is scattered via reflection, refraction and diffraction phenomena and the intensity of the scattered light, which should be proportional to number of NPs in suspension^28^, can be recorded by standard spectrophotometers.

**Figure 1:**
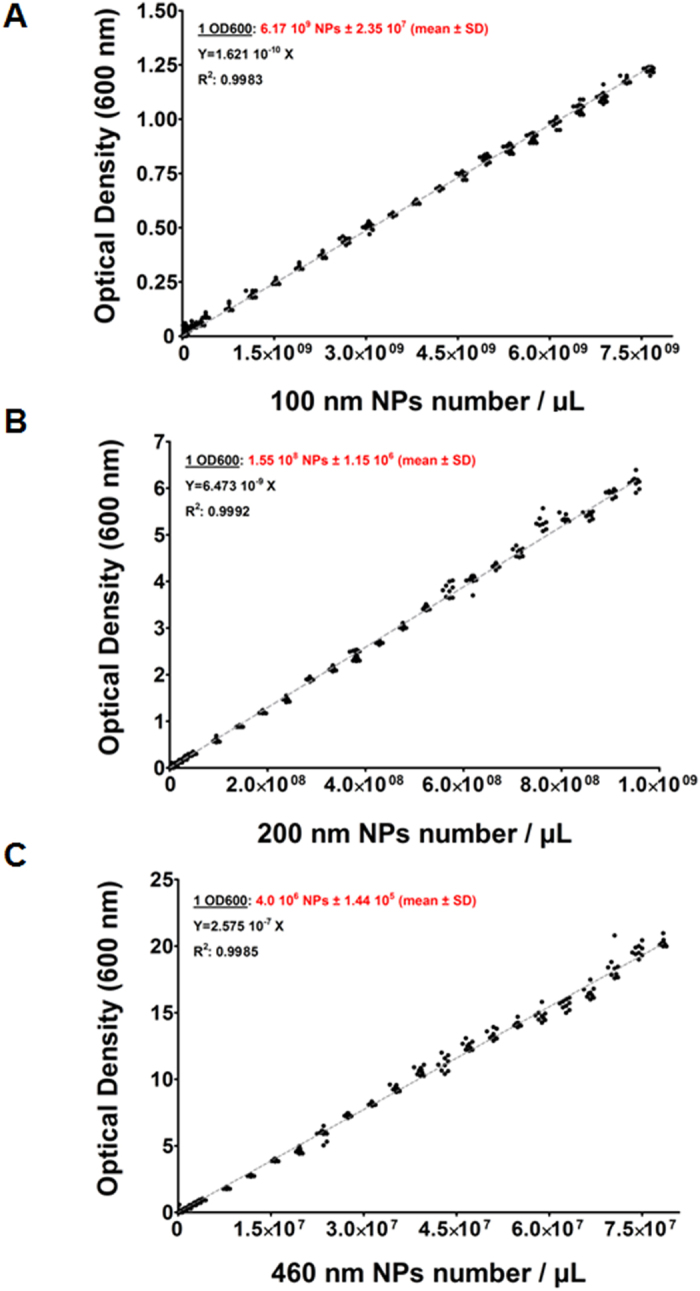
Representation of OD600 measurements vs NPs number/μL. The data were analysed by GraphPad software according to a linear regression straight line equation. (**A**) Calibration curve of 100 nm NPs. (**B**) Calibration curve of 200 nm NPs. (**C**) Calibration curve of 460 nm NPs. 1OD600 value, line equation and correlation factor (r2) are indicated for each NP size. Nine replicates were measured per NPs concentration.

To verify the feasibility of this method, three NP suspensions of different size were used as mother suspensions. Two amino-methyl cross-linked polystyrene NPs of 460 nm and 200 nm with 2% solid content each, corresponding to 3.92 × 10^8^ and 4.77 × 10^9^ NPs/μL for 460 nm and 200 nm respectively, and polystyrene latex nanoparticles of 100 nm with 2% solid content corresponding to 3.82 × 10^10^ NPs/μL. Twenty-six dilutions were then prepared from each mother suspension and used to record their OD600 values after measuring, in parallel, with two different spectrophotometers with different paths: a) a conventional 1 cm path length cuvettes (Eppendorf BioPhotometer) and, b) a 1 mm path length system (NanoDrop Spectrophotometer ND-1000, Thermo Fisher Scientific) (see Supplementary Appendix S1 for details). OD600 values versus NP concentrations (NPs/μL) were plotted in scatter plots and analyzed for each NP size and each spectrophotometer (Supplementary Appendix S1 for suspension concentrations and OD600 values). All scatter plots obtained from each NP size-type with both spectrophotometers fitted linear regression models (r^2^ = 0.99). Fitted lines obtained with the two different spectrophotometers for each size gave equal slopes, being the dynamic range the only difference, while as expected slopes were different between NP sizes (Fig. 1A,B,C) (see Supplementary Appendix S1 for details). Once calibration curves were plotted, the number of NPs per microliter corresponding to one unit of OD600 for each size could be determined (Table 1). The lower the size the bigger the number of NPs per microliter corresponding to one unit of OD600. These differences were expected since bigger NPs scatter more light than the smaller ones.

This straightforward method for preparing standard curves using initial batches of NP suspensions permitted us to estimate, using just 1 microliter of suspensions, the number of eNPs in final batches, which underwent multiple handling procedures. To demonstrate the applicability of the method, a batch of 200 nm amino-methyl cross-linked polystyrene NPs containing 4.83 × 10^9^ NPs/μL were used to produce Cy5-PEG-NPs (Supplementary Fig. S1, S2 and S3). That required three chemical reactions and multiple washing steps. After each chemical reaction, eNPs suspensions were analyzed by spectrophotometry with their OD600 values recorded in triplicate. By applying the new formula presented above for 200 nm NPs, the numbers of particles were calculated (see Supplementary Fig. S2). As anticipated, these data showed that after each chemical reaction with their corresponding washing steps there was always a loss of NPs, decreasing from 4.83 × 10^9^ NPs/μL of the initial batch to 3.86 × 10^9^ Cy5-PEG-NPs/μL of the final one (see reduction of solid content following each chemical reaction in Supplementary Fig. S2). This significant reduction in the numbers of NPs (21% decrease relative to the original concentration) confirms that NPs quantification is necessary after each potentially-perturbing procedure and, thereby, the applicability of a straightforward method of determining NPs numbers, such as this one. We therefore recommend that the number of nanoparticles added to cell cultures should become the parameter of choice for cell-based applications.

In order to put in practice the new parameter, we designed a protocol to determine the ratio of number of cells *versus* number of NPs required for an efficient and controllable “nanofection” (cellular uptake of nanoparticles). We reasoned that nanofection could be analogous to an infection process by nanosized infective agents such as viruses and phages, as in both cases smaller particles (either NPs or infectious agents) get into bigger ones (cells). Based on that analogy we decided to name that ratio as “multiplicity of nanofection” (MNF), as it could be equivalent to the common multiplicity of infection (MOI) term. MOI is the ratio of infectious agents to cells and the key parameter for quantifying the infectivity of a certain agent. For this purpose, it was essential to know the number of NPs per microliter and therefore ideal to demonstrate the applicability of the method presented here.

**Figure 2 f2:**
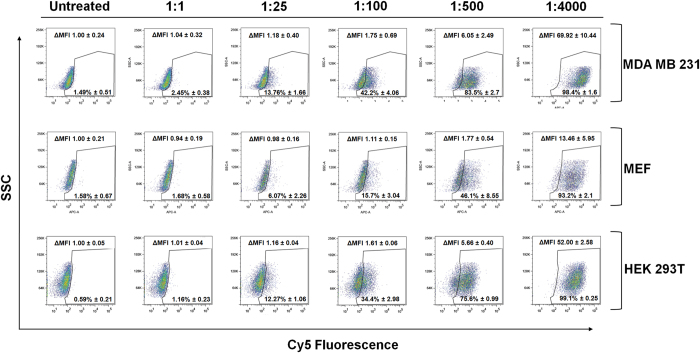
Representative flow cytometry dot plots obtained with MDA MB 231, MEF and HEK 293T incubated with 1:1, 1:25, 1:100, 1:500 and 1:4000 cell to Cy5-PEG-NPs ratios. SSC, side scatter, cellular complexity. Percentages of nanofected cells and increment of median fluorescence intensities (ΔMFI) are expressed as mean ±S.D.

Given that most studies support that the optimal NP size range for *in vivo* applications goes from 10 nm to 250 nm^29^, the previously prepared 200 nm Cy5-PEG-NPs were used to perform these studies (Supplementary Fig. S1). We interrogated different ratios of Cy5-PEG-NPs added to cells to calculate the MNF value of three different cell lines, (i) MDA MB 231 mammalian breast cancer cell line, (ii) mouse embryonic fibroblast (MEF) and (iii) HEK 293T embryonic kidney fibroblast. Cy5-PEG-NPs were incubated with the three cell lines during 24 h using ratios ranging from 1:1 to 1:4000 (cells/Cy5-PEG-NPs). Following that time, cells were washed, trypsined, and analyzed by flow cytometry. Figure 2 shows FACS data represented in dot plots which were used to determine the percentage of cells which were nanofected by Cy5-PEG-NPs (Supplementary Appendix S2, cytometry data). Looking at these results was evident that different cells lines present different MNF values.[Fig f2]

**Figure 3:**
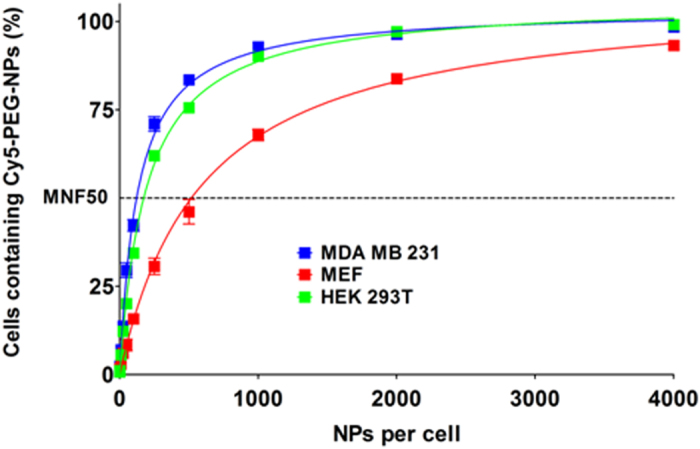
Analysis of Cy5-PEG-NPs cellular uptakes by MDA MB 231, MEF and HEK 293T. Cy5-PEG-NPs, at different ratios per cell, were incubated with MDA MB 231 (Blue), MEF (Red) and HEK 293T (Green) cell lines and analyzed by flow cytometry. Percentage of cells containing Cy5-PEG-NPs versus cell to Cy5-PEG-NPs ratio is displayed. The data (mean ±S.D.) are represented as hyperbola model to study the saturation of cellular uptake behaviour. MDA MB 231, r2 = 0.9927; MEF, r2 = 0.9906; HEK 293T, r2 = 0.9978. MNF50: Multiplicity of nanofection fifty; values indicated in the text.

In human breast carcinoma cell line MDA MB 231, nanofected cells were detected from the lowest ratio (1:1 Cell/Cy5-PEG-NPs), showing a more “permeable” behaviour to be nanofected than the other two cell lines (Fig. 3). The percentage of Cy5 positive cells increased gradually from the lowest ratio up to 1:1000 ratio (Cell/Cy5-PEG-NPs), point at which percentage saturation was reached (Fig. 3). HEK 293T cell line presented a similar uptake profile than MDA MB 231, even though it could be observed a slightly higher resistance to be nanofected than MDA MB 231. In fact, in both cases, at a 1:1000 ratio, percentage saturation was reached with 100% of cells being nanofected. However, MEF cell line showed a clear slower rate of nanofection, being statistical significant the differences with the other cell lines from 1:25 ratio to the highest tested ratio, 1:4000 (Fig. 3, Supplementary Fig. S4 and Supplementary Appendix S2, percentage comparison). Indeed, the 100% of nanofected population was not reached at any ratio used in this study, confirming the MEF resistance to be nanofected (Fig. 3).

To further describe the uptake capability of cell lines, we propose a new index, MNF_50_, defined as the number of NPs capable of nanofecting 50% of the cell population. Percentages of nanofected cells versus ratio were fitted to a hyperbola equation model (Fig. 3) so that the 50% of positive cell populations given by the curve is considered as the MNF_50_ index. For MDA MB 231 cell line MNF_50_ index was established as 130.5 Cy5-PEG-NPs ± 5.1. MNF_50_ value was slightly higher for HEK 293T cell line, 193.4 Cy5-PEG-NPs ± 4.3, and much higher, 598.6 Cy5-PEG-NPs ± 30.6, for MEF cell lines, confirming their poor nanofection (Fig. 3 and Supplementary Appendix S2, percentage comparison).

**Figure 4:**
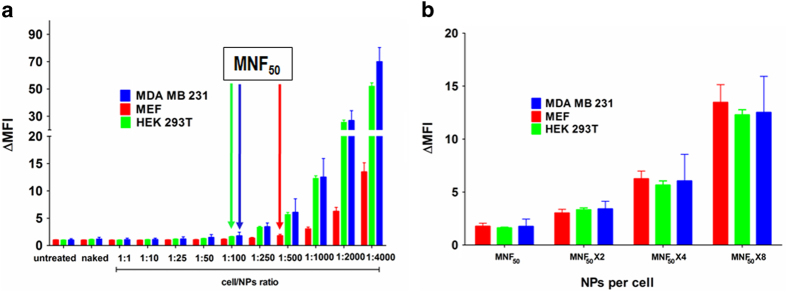
Increment of median fluorescence intensity (ΔMFI) versus cell to Cy5-PEG-NPs ratio. (**a**) Bar representation to compare ΔMFI between MDA MB 231 (Blue), MEF (Red) and HEK 293T (Green) cell lines. Arrows (with respective colors for each cell line) indicate MNF50 (Multiplicity of nanofection fifty). Results are expressed as mean ±S.D. Statistical significance was determined by Bonferroni’s multiple comparison test between the same treatments of the different cell lines (see Supplementary Appendix S2 for details). (**b**) ΔMFI values when cell to Cy5-PEG-NPs ratios for each cell line corresponded to their respective MNF50, MNF50 × 2, MNF50 × 4 and MNF50 × 8.

Besides the MNF_50_ index, a deeper study of the median of fluorescence intensity increments (ΔMFI = MFI sample/MFI untreated) of Cy5-positive cells gave more information about uptake capability of the different cells lines (Fig. 4A). As we expected, following nanofection a wide spread population is observed showing that there is certain heterogeneity from cell to cell (Fig. 2). However, ΔMFI analysis considers this heterogeneity. In this study, these data reveals that the larger number of nanoparticles the greater cellular uptake and although cells are nanofected, nanoparticles uptake continues (Fig. 4A). ΔMFI analysis showed a surprising data. From the closest ratio to their MNF_50_ (1:100 ratio in MDA MB 231 and HEK 293T, and 1:500 ratio, in MEF), the increase of the ΔMFI is doubled when the NPs number are doubled, something which is not observed when ratios lowers than their MNF_50_ are used. Importantly, this effect is the same for the three cell lines (Fig. 4B and Supplementary Fig. S5). Therefore, when MNF_50_ is reached, the nanofection rate appears to become constant, linear and proportional to the number of nanoparticles used with cell lines presenting similar behaviour. Therefore, knowing the MNF_50_ for each cell line against specific eNPs might be crucial to tailor assays and to target specific cell types. These data support the need of using number of particles as a key parameter for cellular-based assays using NPs in order to report number of cells *versus* number of NPs.

**Figure 5:**
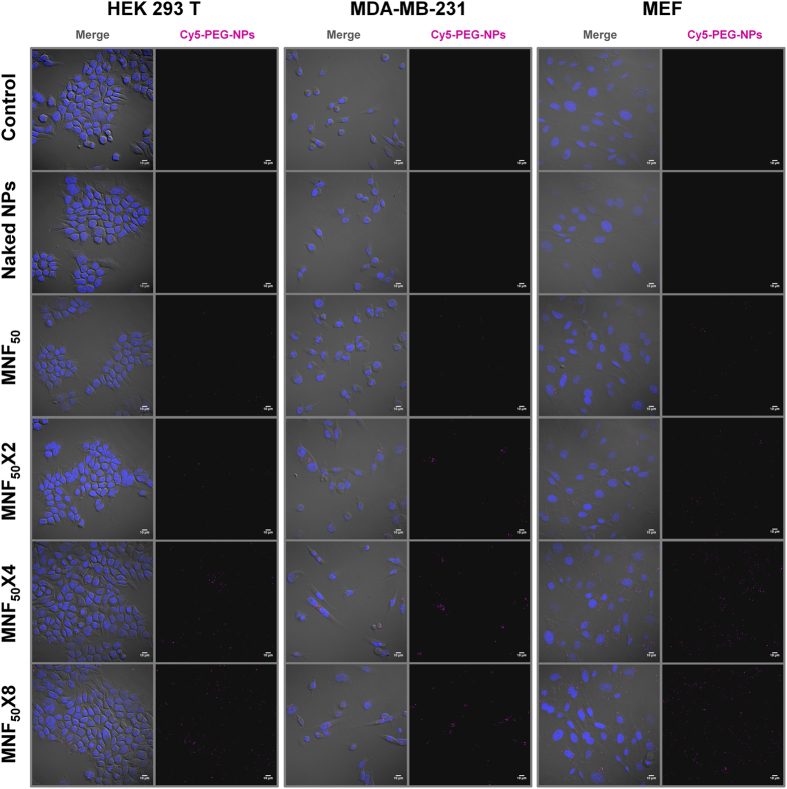
Confocal fluorescence microscopy to analyze Cy5-PEG-NPs cellular uptakes by MDA MB 231, MEF and HEK 293T. Each cell line was incubated with the amount of Cy5-PEG-NPs corresponding to their respective MNF50, MNF50 × 2, MNF50 × 4 and MNF50 × 8 and analyzed by confocal fluorescence microscopy. Untreated cells and cells incubated with naked NPs were used as controls. Left panel: Merge, composition of the three recorded channels (DIC–Differential interference contrast–; blue, DAPI –nuclei–; and red, Cy5-PEG-NPs). Right panel: red channel, Cy5-PEG-NPs. Scale bar, 10 μm.

In order to know if this new index of MNF_50_ can be calculated by other techniques, the phenomenon of nanoparticles internalization from the MNF_50_ was studied by confocal fluorescence microscopy (Fig. 5). The experiment was performed in the previously tested cell lines ranging from the MNF_50_ to MNF_50_ × 8, being monitored in parallel by flow cytometry to confirm previous results. The images confirmed nanoparticles intracellular localization and also that nanofected populations increased when the number of added eNPs per cell were doubled. The analysis of mean fluorescence intensity increment per cell revealed a comparable increase but not identical to those obtained by flow cytometry (Supplementary Table S2). These differences were expected as this type of confocal microscopy is a qualitative technique for single-cell measurements allowing analyze small amount of cells (in contrast to flow cytometry)^30^. These data suggest the method of choice to determine the MNF_50_ index is the flow cytometry.

## Conclusion

**Figure 6:**
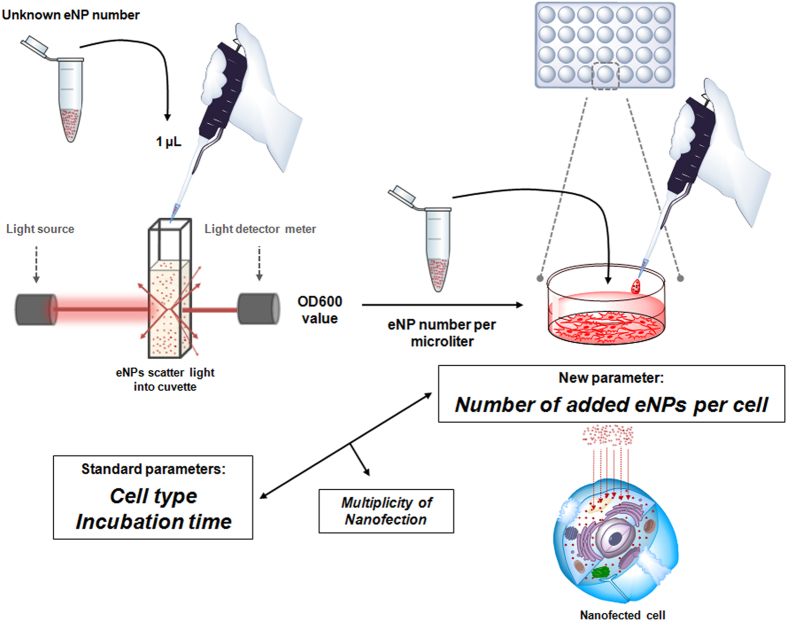
Schematic illustration of the Spectrophotometric Method to Determine Nanoparticle Concentration and its application to cell-based assays based on nanoparticles.

To conclude, a methodology to estimate number of nanoparticles per volume in a quick, low cost and accessible manner is reported. The method can be implemented in any research laboratory, as it just requires a simple spectrophotometer. Using tiny volumes of the suspension, this methodology can be used to calculate the number of ENPs found in final batches which underwent multiple handling processes during preparation steps. This calculation facilitates introducing the number of cells versus number of NPs ratio as parameter to report and standardized protocols for cell-based assays (Fig. 6). By applying this ratio, MNF_50_ index, which gives the number of NPs per cell needed for having the 50% of cells nanofected within a specific time (24 h for this study), is also presented as a new parameter. MNF_50_ indexes were calculated for two human cell lines, a breast cancer line and an embryonic fibroblast and for a mouse embryonic fibroblast line, allowing for the determination of which cell line is more susceptible to be nanofected by a specific NP type. Finally, analyses of ΔMFI values showed that, once cells are 50% nanofected, eNPs are still uptaken by cells following a lineal increment in all cell lines tested as the number of NPs is doubled. This demonstrates that by using number of NPs per cell it is possible to quantify different cellular behaviours in assays using nanodevices.

Based on these data we believe that implementation of this method to calculate the number of nanoparticles per volume will significantly improve the efficiency and reproducibility of any nanoparticle-based experimentation. In particular, this new methodology has generated a new parameter -*number of nanoparticles per cell*- that open a plethora of applications in cell-based assays because ensure the reproducibility and exhaustive control of the assay conditions allowing the comparison between eNPs from different batches. Future work will be focussed on the implementation of this approach to assess nanotechnology-based drug delivery systems as, using number of added eNPs per cell allow defining doses of therapeutic cargoes which are delivered per experimental condition in a robust manner.

## Experimental Section

### Approximation method to calculate nanoparticles concentration based on turbidimetry techniques

The approximation method to calculate NPs concentration was developed using marketed polystyrene NPs in three different size formats (Two amino-methyl cross-linked polystyrene NPs of 200 and 460 nm, purchased from NanoGetic S.L, Granada, Spain and polystyrene latex nanoparticles of 100 nm, purchase from IKERLAT polymers, Lasarte-Oria, Spain.). Several NPs suspensions at different concentrations per microliter were prepared from an initial batch of known concentration according to manufacturer’s specifications (see Supplementary Appendix S1). The optical density at 600 nm (OD600) was recorded three times in three different asssays per suspension using two different spectrophotometers: EppendorfBioPhotometer (with path length of 10 mm cuvettes), and NanoDrop Spectrophotometer ND-1000, Thermo Fisher Scientific (1 mm path length system), OD600 data from NanoDrop Spectrophotometer were corrected by factor (x10) (see Supplementary Appendix S1). The graphs and analysis of data were fitted a straight line with linear regression constrain to 0 using GraphPad Software.

### Cell Culture

Three different cell lines were used in the eNPs uptake study. MDA-MB 231 cell line was cultured in RPMI (Gibco) supplemented with 10% (vol/vol) FBS (Gibco), 1% L-Glutamine (Gibco) and 1% Penicillin/Streptomycin (Gibco). MEF (mouse embryonic fibroblasts) cell line and HEK 293T (embryonic kidney fibroblast) were cultured in DMEM (Gibco) supplemented with 10% (vol/vol) FBS, 1% L-Glutamine and 1%Penicillin/Streptomycin (Gibco). All cell lines were grown in a humidified incubator at 5% CO_2_ and 37° C.

### Preparation of 200 nm Cy5-PEG-NPs

PEGylated 200 nm NPs with Fmoc amine protecting group were coupled to Cy5 dye (see Supplementary Fig. S1). Briefly, amino-methyl cross-linked polystyrene (PS) 200 nm NPs were coupled with Fmoc-1-amino-4,7,10-trioxa-13-tridecamine succinic acid using standard HOBt (1-hydroxybenzotriazole)/DIC (1,3-Diisopropylcarbodiimide) chemistry. Fmoc protected 200 nm NPs were centrifugated (13000 rpm), washed three times with dimethylformamide (DMF) before deprotecting the Fmoc group with 20% piperidine in DMF with three consecutive treatments, 20 min each, at room temperature and shaken at 1400 rpm. Fmoc deprotected 200 nm NPs were then washed three times with DMF and coupled to Cy5. For the coupling, NPs were resuspended in DMF with DIPEA and commercially available Cy5 NHS ester (lumiprobe) was added. The coupling was performed overnight at room temperature and at 1400 rpm. Finally, Cy5-PEG-NPs were two times washed two times with DMF, two times washed with water and finally resuspended in water.

The effectiveness of the Cy5 conjugation was checked by flow cytometry with a FACSCanto II flow cytometer (Becton Dickinson & Co., NJ, USA) and by fluorescence microscopy with Confocal Scanning Microscope Zeiss LSM 710. Dynamic Light Scattering (DLS) and Zeta potential were measured on a Zetasizer Nano ZS ZEN 3500 in molecular biology grade water in a disposable sizing cuvette for size measurements or clear disposable zeta cuvette for zeta potential measurements (Supplementary Table S1).

### Cy5-PEG-NPs Uptake Study by flow cytometry

Cells were washed with phosphate buffered saline (PBS 1X), detached with trypsin/EDTA (0.25%, phenol red) (Gibco), counted and diluted with media to a final concentration of 10^5^ cells per mL. 500 μL of each cell line suspension were plated in 24 well plates (Nunc) and incubated for 12 h. Then, media was replaced with a fresh media mixture containing specific number of Cy5-PEG-NPs to study different cells/Cy5-PEG-NPs ratios: 1/1; 1/10; 1/25; 1/50; 1/100; 1/250; 1/500; 1/1000; 1/2000 and 1/4000. Cy5-PEG-NPs number was measured using the method describe above. As control were used unconjugated NPs (at ratio 1/4000, cell/NPs), referred as naked NPs, and cells without nanoparticles treatment. After 24 h incubation with Cy5-PEG-NPs, the media was aspirated and cells were washed with PBS 1X, and detached with Trypsin-EDTA (0.25%) at 37° C for 5 minutes. Samples were analyzed via flow cytometry with a FACSCanto II flow cytometer (Becton Dickinson & Co., NJ, USA). Each experiment was done in duplicate per ratio and repeated three times per cell line.

Dot plots and cytometry statistics were obtained using FlowJo software (Percentage of positive population and median of fluorescence intensity; see Supplementary Appendix S2 for data and significant statistical analysis). Graphs and statistical difference data were performed using GraphPad Software according to the following explanation. Percentage data of cells containing Cy5-PEG-NPs were represented versus cell/Cy5-PEG-NPs ratio in two different graph types. First in a XY representation according to hyperbola equation fitting model to study the saturation Cy5-PEG-NPs profile of the different cell lines and the specific multiplicity of nanofection fifty (number of nanoparticles to obtain 50% of cells containing Cy5-PEG-NPs (nanofected)). Second in a bar representation to establish statistical significant differences by two-way ANOVA Bonferroni’s multiple comparison test comparing the same treatments between different cell lines. Futhermore, median fluorescence intensity (MFI) was exhaustive analyzed comparing the MFI increment (ΔMFI, MFI sample/MFI untreated). Two-way ANOVA Bonferroni’s multiple comparison test was applied to study statistical significance.

### Cy5-PEG-NPs Uptake Study Confocal fluorescence microscopy

Cells were washed with phosphate buffered saline (PBS 1X), detached with trypsin/EDTA (0.25%, phenol red) (Gibco), counted and diluted with media to a final concentration of 10^5^ cells per mL. 500 μL of each cell line suspension were seeded onto glass poly-L-lysine-coated coverslips (Neuvitro) in 24 well plates (Nunc) and incubated for 12 h. Then, media was replaced with a fresh media mixture containing the amount of Cy5-PEG-NPs to reach MNF_50_, MNF_50_ × 2, MNF_50_ × 4 and MNF_50_ × 8 values for each cell line. Cy5-PEG-NPs number was measured using the method describe above. Unconjugated NPs, referred as naked NPs (at ratio 1/4000, cell/NPs), and cells without nanoparticles treatment were used as controls. Following 24 h incubation with Cy5-PEG-NPs, the media was aspirated and cells were washed with PBS 1X, and fixed in 4% paraformaldehyde (PFA) at room temperature for 30 minutes. Fixed cells were washed with PBS 1X and coverslips were mounted with ProLong Gold antifade mountant with DAPI (Life technologies). Image acquisition was performed with Confocal Scanning Microscope Zeiss LSM 710 using a 63X objective and Zen 2012 software. Each experiment was done in duplicate per ratio and repeated three times per cell line.

Images were analyzed using ImageJ software (Research Services Branch, National Institute of Mental Health, Bethesda, Maryland, USA) ( http://rsb.info.nih.gov/ij/index.html). Cy5 mean fluorescence intensity of each image was determined on the specific filtered channel selecting the command “Measure” from the *analyze* menu. To count objects, nucleus (cells), the specific channel (DAPI) of each image were subjected to the threshold function and then counted selecting the command “Analyze particles” from the *analyze* menu using the size (μm^2^) interval “4-Infinity”. Cy5 mean fluorescence intensity was normalized versus cell number and it was analyzed comparing the mean fluorescence intensity (Mean fluorescence intensity sample/mean fluorescence intensity untreated).

## Author Contributions

J.D.U.B and V.C.C contributed equally. J.D.U.B and V.C.C prepared Engineered Nanoparticles (eNPs). J.D.U.B, V.C.C and P.A.M carried out biological experiments. J.D.U.B and V.C.C analyzed raw data and performed statistical analysis. J.D.U.B introduced Multiplicity of Nanofection fifty (MNF50). SP, J.D.U.B and R.M.S.M contributed to design biological experiments. J.D.U.B, J.J.D and R.M.S.M interpreted the biological data and wrote the paper. J.D.U.B and R.M.S.M directed the work. The manuscript was written through contributions of all authors. All authors have given approval to the final version of the manuscript.

## Additional Information

**How to cite this article**: Unciti-Broceta, J. D. *et al.* Number of Nanoparticles per Cell through a Spectrophotometric Method - A key parameter to Assess Nanoparticle-based Cellular Assays. *Sci. Rep.* **5**, 10091; doi: 10.1038/srep10091 (2015).

## Supplementary Material

Supplementary Information

Supplementary Appendix S1

Supplementary Appendix S2

## Figures and Tables

**Table 1:** Number of NPs per microliter corresponding to one unit of OD600.

**NP sizes**	**Number of NPs per microliter corresponding to one unit of OD600 (mean ±SD)**
460 nm	4 × 10^6^ NPs/μL ± 1.44 × 10^5^
200 nm	1.55 × 10^8^ NPs/μL ± 1.15 × 10^6^
100 nm	6.17 × 10^9^ NPs/μL ± 2.35 × 10^7^
