# Supplementary material for: Number of Nanoparticles per Cell through a Spectrophotometric Method - A key parameter to Assess Nanoparticle-based Cellular Assays
Source: Sci Rep. 2015 May 15;5:10091. doi: 10.1038/srep10091 (PMC4432369; doi:10.1038/srep10091)
Supplement: Supplementary Information [file srep10091-s1.pdf]

Number of Nanoparticles per Cell through a Spectrophotometric Method - A key parameter to Assess Nanoparticle-based Cellular Assays

Juan D. Unciti-Broceta*†[ab], Victoria Cano-Cortés†[a], Patricia Altea-Manzano[a], Salvatore Pernagallo[c], Juan J. Díaz-Mochón[a], Rosario M. Sánchez-Martín*[a]

[a] *Pfizer - Universidad de Granada - Junta de Andalucía Centre for Genomics and Oncological Research (GENYO), Parque Tecnológico Ciencias de la Salud (PTS), Avenida de la Ilustración 114, 18016 Granada, Spain.*

Departamento de Química Farmacéutica y Orgánica. University of Granada, Campus de Cartuja s/n, 18071 Granada, Spain.

[b] *NanoGetic S. L. Parque Tecnológico Ciencias de la Salud (PTS), Avenida de la Innovación 1, Edificio BIC, 18016 Armilla – Granada (Spain).*

[c] *DestiNAGenomica S.L. Parque Tecnológico Ciencias de la Salud (PTS), Avenida de la Innovación 1, Edificio BIC, 18016 Armilla – Granada (Spain).*

† These authors contributed equally to this work.

* To whom the correspondence should be addressed. E-mail: rmsanchez@ugr.es; juandiego.unciti@genyo.es

Supplementary Figure S1

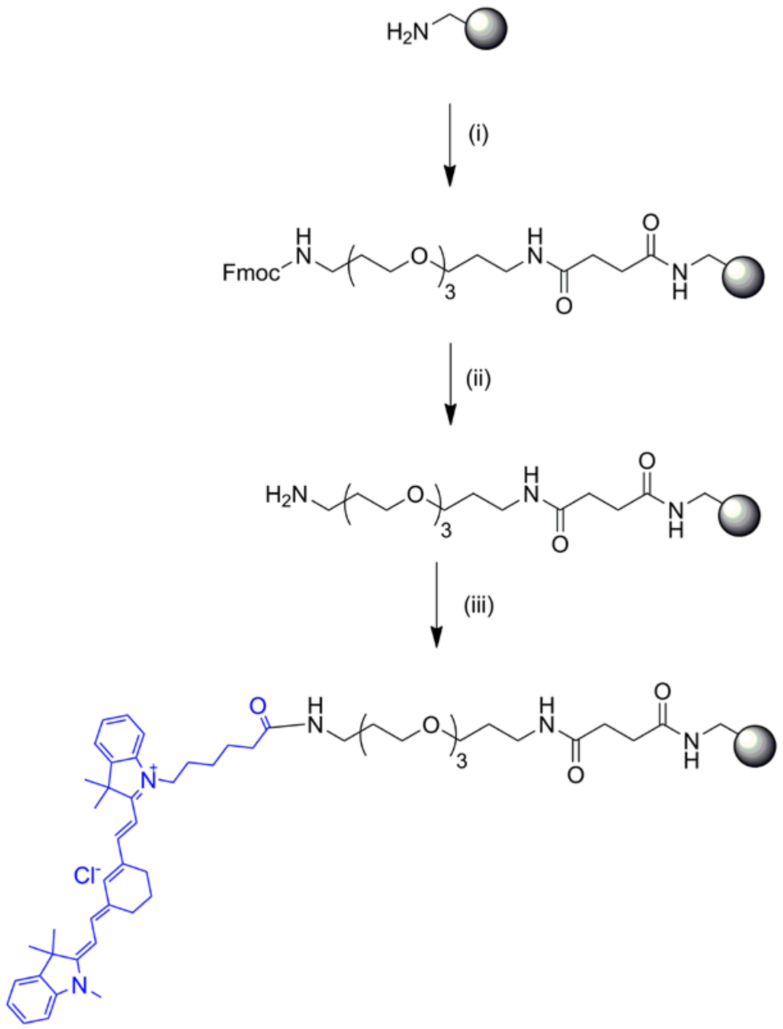

Reagents and Conditions: (i) Fmoc-4,7,10-trioxa-1,13-tridecanediamine succinamic acid (PEG), Oxyma, DIC, DMF, 60 ºC, 1400rpm, 2h, 100%; (ii) 20% piperidine in DMF; (iii) Cy5-NHS, DIPEA, DMF, RT, 1400rpm, O/N, 100%.

Supplementary Figure S1. Reagents and conditions: (i) Fmoc-1-amino-4,7,10-trioxa-13-tridecamine succinic acid (PEG), oxyma, DIC, DMF, 60° C, 1400 rpm, o7n, 100%. 2h, 100%; (ii) 20% piperidine in DMF; (iii) Cy5-NHS, DIPEA, DMF, RT, 1400 rpm. Abbreviations: PS, polystyrene; PEG, polyethylene glycol; NP, nanoparticle.

Supplementary Figure S2

	NP/µL	SD
Original batch	4.83e+009	6.45e+007
PEG Fmoc conjugation	4.26e+009	5.59e+007
Fmoc deprotection	4.04e+009	3.23e+007
Cy5 conjugation	3.86e+009	1.55e+007

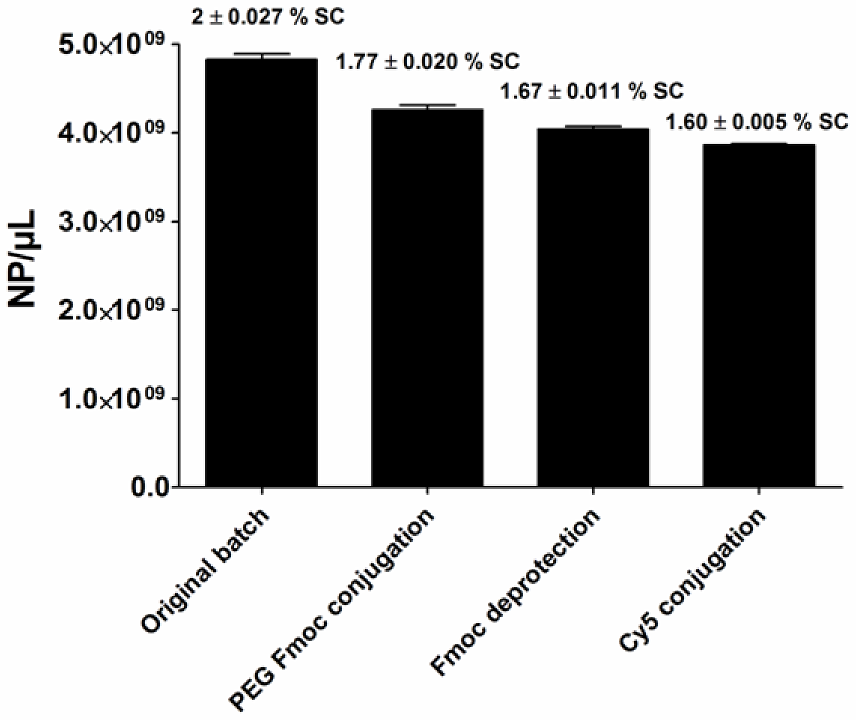

Supplementary Figure S2. NP number per µL calculated by the described method after in each step of NP engineering. Columns represent the mean of three measurements and error bar indicates standard deviation. Numerical data are indicated in the table. Solid contain was calculated considering the measured NP/µL of the original batch as the 2%.

Supplementary Figure S3

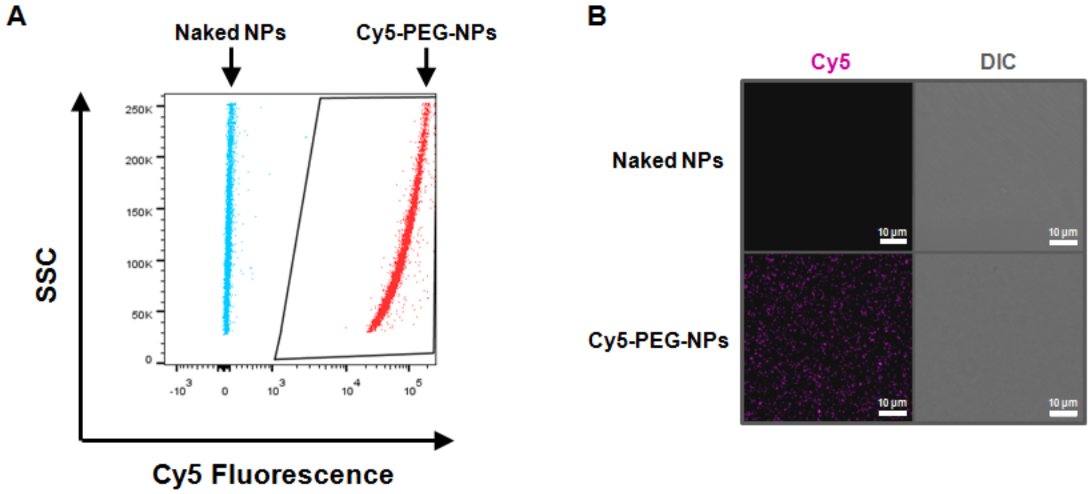

Supplementary Figure S3. Fluorescence Cy5-PEG-NPs characterization. (A) Representative overlay dot plot obtained after flow cytometry analysis of naked NPs (blue) and Cy5-PEG-NPs (Red). *SSC*, side scatter. (B) Confocal fluorescence microscopy of naked NPs and Cy5-PEG-NPs. *DIC*, Differential Interference Contrast. *Scale bar*, 10 µm.

Supplementary Figure S4

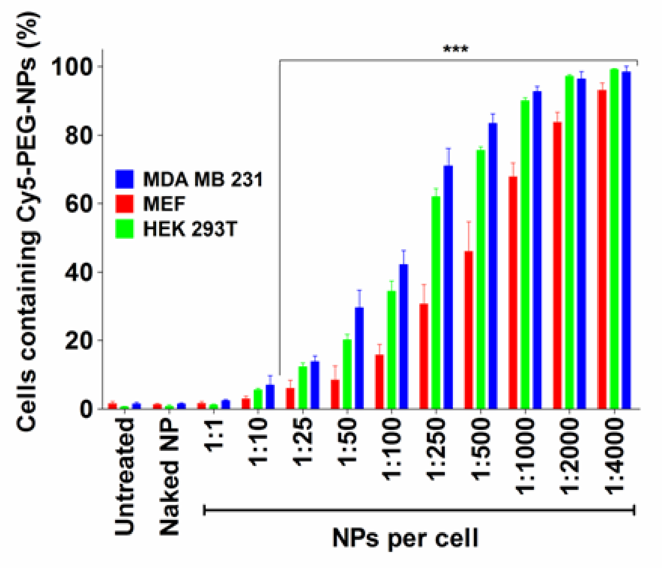

Supplementary Figure S4. Analysis of Cy5-PEG-NPs cellular uptakes by MDA MB 231, MEF and HEK 293T. Cy5-PEG-NPs, at different ratios per cell, were incubated with MDA MB 231 (Blue), MEF (Red) and HEK 293T (Green) cell lines and analyzed by flow cytometry. Percentage of cells containing Cy5-PEG-NPs versus cell to Cy5-PEG-NPs ratio is displayed in a bar representation to compare cellular uptake. Results are expressed as mean ± S.D. *** Statistical significance differences (see Supplementary Appendix S2 for details) were determined by two-way ANOVA Bonferroni's multiple comparison test between the same treatments of the different cell lines.

Supplementary Figure S5

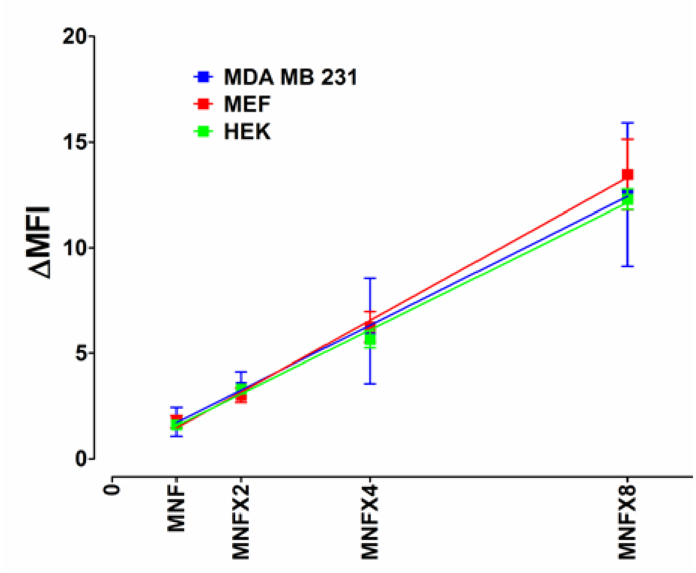

Supplementary Figure S5. Increment of median fluorescence intensity (ΔMFI) versus cell to Cy5-PEG-NPs ratio. Representation to compare ΔMFI between MDA MB 231 (Blue), MEF (Red) and HEK 293T (Green) cell lines when cell to Cy5-PEG-NPs ratios corresponded to their respective MNF_{50}, MNF_{50}X2, MNF_{50}X4 and MNF_{50}X8. Results are expressed as mean ± S.D.

Supplementary Table S1. Particle characterization.

	Particle size (nm)	PDI	Zeta potential (mV)
Amino-methyl PS	222.1	0.002	32.3
PEG Fmoc conjugation	220.9	0.015	-38.9
Fmoc deprotection	221.8	0.017	-31.4
Cy5-PEG-NPs	221.9	0.008	-23.9

PDI = Polidispersity Index

Supplementary Table S2. Confocal mean fluorescence intensity increment per cell.

	MDA MB 231		MEF		KEK 293T	
	Mean	± SEM	Mean	± SEM	Mean	± SEM
Untreated	1	± 0.05	1	± 0.03	1	± 0.02
Naked NPs	1.14	± 0.08	1.95	± 0.10	1.11	± 0.04
MNF_{50}	1.73	± 0.13	2.53	± 0.17	2.11	± 0.29
$MNF_{50}X2$	2.76	± 0.31	4.86	± 0.24	2.66	± 0.45
$MNF_{50}X4$	4.06	± 0.53	9.11	± 0.44	3.72	± 0.22
$MNF_{50}X8$	5.49	± 0.59	11.18	± 1.09	6.65	± 0.62

Supplementary Table S2. Confocal mean fluorescence intensity increment per cell (mean fluorescence intensity sample/mean fluorescence intensity untreated). Cy5 channel mean fluorescence of the images was measured using ImageJ software. Results are expressed as mean ± S.E.M.

Supplementary Note S1

$$\text{Number of NPs per mL} = \frac{6 \times 10^{10} \times \text{Solid content } (\%) \text{ x } \rho_{\text{suspension}}}{\pi \times \rho_{\text{particles}} \times d^3}$$

Where: Solid content (%) = weight % (g per 100 mL)

$$\rho_{suspension} = \frac{100 \times \rho_{particles}}{Solid\ \ content\ \ (\%) \times (1 - \rho_{particles}\ ) + (100 \times \rho_{particles}\ )}$$

$\rho_{particles} = 1\,{}^{g}/_{dm^3}$ for polystyrene particles
